# Blockchain and supply chain finance: a critical literature review at the intersection of operations, finance and law

**DOI:** 10.1007/s42786-022-00040-1

**Published:** 2022-05-09

**Authors:** Ilias Ioannou, Guven Demirel

**Affiliations:** 1 Centre for Commercial Law Studies, Queen Mary University of London, 67-69 Lincoln’s Inn Fields, London, WC2A 3JB UK; 2 School of Business and Management, Queen Mary University of London, The Bancroft Building, Mile End Road, London, E1 4NS UK

**Keywords:** Blockchain, Distributed ledger technology, Smart contract, Supply chain finance, Trade finance, Fintech

## Abstract

In the current environment, where the Covid-19 pandemic has exposed the vulnerabilities of the incumbent paper-based trade and supply chain finance systems, digital transformation pledges to alleviate the friction on international trade. Here, we provide a timely review of state-of-the-art industry applications and theoretical perspectives on the use of blockchain as the medium toward digitalisation for supply chain finance systems. We argue that blockchain technology has an innovation promoting role in supply chain finance solutions through reducing inefficiencies and increasing visibility between different parties, which have hitherto constituted the main challenges in this sphere. Based on a review of the academic literature as well as an analysis of the industrial solutions that have emerged, we identify and discuss the financial, operational and legal challenges encountered in supply chain financing and the promise of blockchain to address these limitations. We discuss the bottlenecks as well as the benefits of blockchain and identify some necessary conditions required for the emergence of blockchain-enabled trade and supply chain financing, such as the establishment of co-opetition among supply chain actors, integration with IoT systems for data quality, and reform of regulatory and legal frameworks. We conclude by identifying promising research directions about the implementation process, inviting further research into the transformation of business models toward a more collaborative nature.

## Introduction

An important but still relatively undervalued use case of blockchain technology is Supply Chain Finance (SCF). Up to $80\%$ of international trade transactions require trade and SCF to provide liquidity and risk mitigation [42]. The financing of trade transactions was estimated by the European Commission to be worth USD 10 trillion in 2017 alone [98]. It includes both various methods for the discharge of the payment obligation as well as techniques and practices for the optimisation of the working capital invested in supply chain transactions, such as receivables purchase techniques or accounts payable-centric finance. However, the ingrained reliance of trade and supply chain financing on paper-based documentation has driven up costs and caused inefficiencies. Fragmented processes, discordance of regulations, and the increased risk of fraud contribute together to a USD 1.5 trillion supply-demand gap in the financing of trade [2], which, if left unresolved, is expected to exceed USD 2.4 trillion by 2025 [155].

While SCF is difficult to obtain for many stakeholders in an ordinary business environment, the ongoing pandemic and global recession magnify the existing pain points and barriers in SCF and pose new ones of unprecedented scale [73]. Most of the problems being faced today originate from the paper medium used in SCF and relate to the delivery and the handling of physical documents, the lack of staff, the inability to print, and business closures due to lockdown restrictions [96, 108]. Moreover, the necessity of validating the originality of documents and the legal matters that emanate from jurisdictions requiring wet-ink signed payment obligations and transport documents have challenged the industry’s capacity to deal with this unrivalled disruption on a global scale [73]. The existing gap in the financing of trade, which according to the International Financing Corporation of the World Bank Group is now anticipated to exceed USD 4 trillion [74, 121, 139], is set to double.

In essence, SCF techniques aim to reliably establish the creditworthiness of the buyer of goods and approve that the sellers of goods have manufactured and shipped them [9]. The past five years have witnessed a proliferation of research, initiatives and discussions regarding blockchain as the medium toward digitalisation of the supply chain [85–87, 120]. Significant advancements have been made and the obstacles are gradually being removed, improving the efficacy of information flow in the supply chain and increasing the flexibility of the financial supply chain [17, 33, 48], both of which run alongside the physical supply chain [157]. The aim of this review is to complement the literature’s interest in the usability of blockchain in international trade and to identify the main drivers and challenges of digital transformation within the trade and supply chain finance industry.

In this context, there are several reasons for undertaking a critical literature review on the interface of blockchain and supply chain finance. First, the industry has expressed a keen interest in adopting new technologies and SCF is well oriented in innovative financing solutions. Second, the growing body of academic literature [23] and the emerging range of supply chain financing systems deserve a review, which will illuminate the benefits and the limitations of blockchain SCF procedures. Third, whilst previous research focuses either on blockchain implementation in supply chain operations [15, 160] or on analysing supply chain financing solutions [10, 54, 158], this is the first specialised review combining the literatures on both blockchain and SCF, and it uncovers knowledge from companies that are pioneering blockchain in their SCF products.

We focus on three research questions in providing an overview of the research on blockchain technology in SCF:

RQ 1: What are the key operational, financial, and regulatory barriers holding back innovation in SCF?
RQ 2: How can blockchain technology support digital SCF integration and could it give rise to new and innovative SCF solutions?
RQ 3: What are the implementation challenges of blockchain adoption in SCF?

The remainder of this paper is organised as follows. Section 2 summarises the basic concepts related to SCF and blockchain technology, providing an account of the various SCF techniques as well as an introduction to blockchain foundations. The findings of our analysis of the literature are provided in Sect. 3, revealing the state-of-the-art developments in both theory and practice. To that end, Sect. 4 discusses the insights the literature offers for the barriers and pain-points of SCF systems, the ways blockchain can alleviate these, and the implementation challenges for the adoption of blockchain-based SCF systems. This section goes on to identify promising research directions using a cross-disciplinary perspective, and it concludes by presenting the limitations of this study. The review concludes with a summary of the main contributions in Sect. 5.

## Background

### Supply chain finance

SCF is a micro-finance concept defined as the use of financial instruments, practices, and technologies for optimising the management of the working capital and liquidity tied up in supply chain processes between collaborating business partners [19]. According to Xu et al. [158] and Ali et al. [4], the term was introduced by Stemmler [131], who explained that SCF constitutes an essential part of supply chain management (SCM) and aims to integrate finance with the supply chain operations. The appeal of SCF is to mitigate the payment and performance risks and to concurrently offer to the supplier accelerated receivables and to the buyer protracted credit [23, 54]. It is distinct from trade finance, which is an overarching term describing the financing of trade in general [141] and which is traditionally associated with financing techniques governed by rules published by the International Chamber of Commerce (ICC), such as the Uniform Rules for Collections (URC 522) for Documentary Collections, Uniform Rules for Demand Guarantees (URDG 758) for Guarantees, and the Uniform Customs and Practice for Documentary Credits (UCP 600) for Letters of Credit (L/Cs) [62].

While the financial supply chain usually refers to the discharge of the payment obligation by the buyer upon receipt of evidence of contractual performance by the seller [57], SCF is a more complex notion and scholars have taken a range of different approaches. According to Hofmann [67], SCF is an approach of two or more organisations ‘to jointly create value through means of planning, steering and controlling the flow of financial resources on an inter-organisational level’. Similarly, Pfohl and Gomm [111], define SCF at the inter-company level as the ‘integration of financing processes to increase the value of all participating companies’. A comprehensive literature review dealing with the various definitions of SCF, and its specific solutions, is provided by Gelsomino et al. [54], who identified two major perspectives: financial-oriented, which refers to short-term receivables and payables SCF solutions provided by financial institutions, and supply chain-oriented perspective, which extends SCF scope to include the capitalisation of inventories and financing provided by non-banks [22, 54]. While earlier reviews [54, 158] cover papers from 2000 to 2016, this paper focuses on current developments in the field, specifically blockchain-enabled solutions.

SCF solutions are designed to increase the visibility and the availability of cash and reduce its cost for all supply chain partners [58, 60] with a view to optimise the management of financial flows at the supply chain level [55]. Some scholars focus more on the central role that banks play in SCF [31, 93, 157], defining SCF as the set of products that a financial institution offers to facilitate the management of the material and information flows in a supply chain [21]. Others consider technology an essential component in the SCF scheme, describing it as financial services solutions stemming from technology service providers [36, 89]. In the operations management literature, SCF solutions have been classified with respect to the party that provides the financing, i.e. trade credit, buyer finance and inter-mediated finance [10, 34, 135]. All the aforementioned elements are summarised in a definition suggested by the Global Supply Chain Finance Forum (GSCFF), which describes SCF as ‘the use of financing and risk mitigation practices and techniques to optimise the management of working capital and liquidity invested in supply chain processes and transactions’ [56]. This article will hereinafter build upon this definition and use the relevant terminology suggested by the GSCFF, which applies irrespective of the role or the existence of an intermediary and the specific enabling technology.

At a basic level, SCF consists of receivables purchases (receivables discounting, forfaiting, factoring and receivables securitisation), payables finance (dynamic discounting, reverse factoring and reverse securitisation) and borrowing using trade credit/accounts receivables as collateral (loan or advance against receivables, distributor finance, inventory finance and pre-shipment finance) [31]. In Table 1, we provide the most commonly used definitions and the synonyms of the SCF techniques based on the classification recommended in Global Supply Chain Finance Forum [56].

**Table 1:** Categorisation of supply chain finance solutions

Category	Techniques	Definition	Synonyms	References
Receivables Finance	Receivables	In receivables discounting a finance provider buys receivables represented by outstanding invoices from a seller at a discount	Invoice discounting,	[31, 62]
	Discounting		Early payment of receivables	
	Forfaiting	Forfaiting is the without recourse purchase of future payment claims represented by financial instruments or payment obligations, at a discount or at a face value in return for a financing charge	Without recourse financing, Discounting of promissory notes / Bills of Exchange	[54, 165]
	Factoring	In factoring, sellers sell their short-term receivables at a discount to a financer (the factor). Finance providers usually become responsible for collecting the payment of the underlying receivables	Receivables finance, Receivables services, Invoice discounting, Debtor finance	[21, 30, 158]
	Account	Securitisation allows credit to be provided by selling the income producing assets (outstanding invoices) at a discount to a special purpose vehicle company (SPV), which then transforms them in asset-backed securities (ABS) and sells in the capital market	Supplier-led securitisation	[69, 81]
	Receivables		Multi-investor model	
	Securitisation		Supplier-led account receivables securitisation	
Payables Finance	Dynamic	Dynamic discounting is a short-term financing instrument initiated by the buyer, who may utilise its own funds to pay an account payable prior to the due date at a variable discount rate	Payables finance,	[32, 103]
	Discounting		Flexible discounting	
	Reverse	Reverse factoring is provided through a buyer-led program within which a seller is provided with the option of receiving the discounted value of receivables prior to their actual due date and typically at a cost aligned with the credit risk of the buyer	Payables finance,	[55, 95]
	Factoring		Import finance,	
			Post-shipment finance	
	Reverse	While in accounts receivable securitisation the risk is calculated on the performance of the isolated pool of receivables, in reverse securitisation, the credit risk is concentrated on one entity for which risk can be quantified	Buyer-led securitisation,	[54, 69]
	Securitisation		Approved payables securitisation	
Loan or Advance-based Finance	Loan/Advance Against Receivables	Loan or advance against receivables is financing made available on the expectation of repayment from funds generated from current or future trade receivables	Receivables lending, Trade receivables loans, Trade Loans	[22, 56]
	Distributor	Distributor finance is the provision of financing for a distributor to bridge the liquidity gap until the receipt of funds from receivables following the sale	Buyer finance, Dealer finance,	[54, 56]
	Finance		Channel finance	
	Pre-shipment	Pre-shipment finance is a loan provided by a finance provider to a seller who has received the purchase order, usually to cover the working capital needs for the order’s execution, such as materials, wages, or packaging costs	Purchase order financing,	[19, 157]
	Finance		Contract monetisation,	
			Packing credit/finance	
	Inventory	Inventory financing is provided to a buyer or a seller involved in a supply chain for the holding or warehousing of goods (which are used as a collateral)	Loan against inventory,	[45, 68, 92]
	Financing		Warehouse receipt finance	

Despite the variations among these mechanisms, a common feature of all SCF techniques is their need to access and process trustworthy trade data [57, 92]. This is because SCF is an event-driven financing solution in that each intervention in the financial chain is ‘triggered’ by an event in the physical chain [4, 165]. For example, receivables purchase techniques require access to reliable trade documentation which can verify the receivables, such as invoices or e-invoices [57]. Similarly, loan or advance-based techniques require access to data that can evidence the expectation of repayment such as, purchase order confirmations, transport documentation and warehouse receipts, while the trigger event in payables financing solutions is usually proved with the approval of the invoice from the buyer [69]. The coupling of information and material flows enables financers to reduce both the financial and operational risks within the supply chain and mitigate the credit risk [10, 92], thereby enabling capital-constrained firms to access capital sooner and at lower rates [31, 93]. This work investigates how the adoption of blockchain technology increases visibility into reliable trade data and allows businesses to form partnerships and accelerate cash flows throughout the financial supply chain.

### Foundations of blockchain technology

Blockchain is a digital distributed ledger of time-stamped series of data records that is stored on a cluster of computers where no single entity has control, and the information is visible to all parties [52, 137]. Transactions are broadcasted to the network and the full-node participants validate them directly through the operation of a consensus mechanism [7]. The full-node participants or miners validate whether there is a successful delivery from the sender to the recipient and examine the veracity of the signed acknowledgements provided by the intermediate nodes [63]. An encryption method secures data against unauthorised interference to ensure censor-resistance and to safeguard sensitive information [41]. A key aspect of blockchain is its anti-double spending feature, which ensures that a person transferring an asset in the form of unspent transaction outputs/inputs [7] or in the form of a balance within an account [8] cannot transfer the same asset more than once [137].

Blockchains are classified as permission-less (‘public’) and permissioned, in alignment with the extent to which nodes may be involved in the consensus process [52, 164]. In a permission-less blockchain, such as Bitcoin or Ethereum, anyone can run as a pseudonymous full node, make contribution, and receive awards pursuant to the corresponding rules. Permissioned blockchains can be further categorised into private and consortium-based blockchains. Simply put, consortium Blockchains, such as the Hyperledger project, have a governance structure and consensus procedures controlled by pre-set nodes in the system [20]. In private blockchains, which can be built on Hyperledger Fabric [6] or Corda [64], for example, access is controlled by a single organisation [137]. A comparison of key features among different types of blockchain is provided in Chang et al. [25] and Tasca and Tessone [137], who argue that the extent of decentralisation is weaker in permissioned blockchains, but the speed of transaction validation is faster [146]. It is noted that an extensive discussion regarding the differences and the similarities between different blockchains of the same class/type regarding their appropriateness for SCF techniques is, to the best of our knowledge, absent from the literature.

From a technical perspective, blockchain comprises a decentralised data infrastructure employing a cryptographic hash function [45]. It can be considered as an infrastructure layer that runs on top of the internet and which is suitable for recording, tracing, monitoring, and transacting all type of assets on a global scale [149]. The first blockchain application was a data protocol for keeping the chronological records of Bitcoin transactions [105]. Since then, blockchain technology has been hailed as an ingenious innovation with countless possibilities for applications in numerous areas [41, 136]. In this regard, the digitisation of documents and the tokenisation of assets into the blockchain can help dismantle financing barriers and pain points in international trade transactions. In the next sections we will examine how blockchain can address existing inefficiencies in trade and supply chain finance processes based on a detailed review of the extant literature.

### Contribution to the literature

Blockchain technology is a significant high-tech breakthrough that may revolutionise SCF. This paper is one of a few works that endeavour to illuminate the positive disruption caused by blockchain for trade and supply chain finance processes. The review examines the existing research on the subject matter and highlights the identified gaps in the literature. It proposes a re-examination of the subject matter through the prism of foundational concepts and results from supply chain management (SCM), economics, legal analysis and platform theory. The provided practical and theoretical insights can be conducive to reflection by SCF practitioners and serve as a base for future academic studies on blockchain adoption in SCF.

## Current developments in blockchain supply chain finance

This section presents the scientific publications identified through the research protocol outlined in Appendix 1 and the state-of-the-art business developments. Some common themes observed in the literature and in practice are summarised in this section. The areas in which blockchain provides most value to SCF will be explored in the next section.

### Academic literature

Although blockchain is still in its nascence, its capacity for trade and supply chain finance has already been acknowledged in the academic literature, where related value-added activities are being mapped and several implementation systems have been proposed. Bogucharskov et al. [17] have proposed a blockchain prototype of a documentary Letter of Credit (L/C). Similarly, Chang et al. [26] and Tsiulin et al. [144] discuss modern blockchain-supported L/C services built on a consortium blockchain, while Chang et al. [25] recommend the re-engineering of L/Cs via smart contracts, which is argued to improve the performance of the payment process and enhance the overall supply chain efficiency.

Chen et al. [30] leverage blockchain, alongside systems and technologies such as cloud computing and the Internet of Things (IoT), to establish an integrated SCF platform running as-a-service for the automotive retail industry. The platform, called Blockchain auto SCF, provides equal visibility on transactions and collateral custody information to interested parties and collaborates with financial institutions to supply inventory financing and purchase order financing [30]. Yu et al. [162] move beyond the performance analysis of operations under the existing SCF techniques and propose a new model for SCF that enables a platform-based financer to offer the best SCF solutions under different conditions and to optimise service fees and price setting based on the client’s opportunity cost rate for self-guarantee. This is achieved by leveraging reliable information stored in a blockchain that demonstrate to the financer, based on the customer’s operational information, the sufficiency of the credit or assets. The proposed model also enables the customer to mortgage its assets, which can range from raw materials to finished products, and transfer these assets to the financer in case of default, all happening in an integrated manner on the blockchain [162].

In their analysis, Omran et al. [107] describe the use cases of blockchain for reverse factoring and dynamic discounting. Reverse factoring can be optimised because blockchain enables invoice status information to be transferred securely, allowing financiers to offer high-frequency financing services for any transaction value at lower risk [107]. In conjunction with smart contracts, blockchain can improve the access to reliable real-time information and automate decision-making through the integration of financial and informational flows in supply chains [93, 157]. That way, the risk premium of an early payment financing proposal can be continuously adjusted at each step of the material flow [107]. Hofmann et al. [69] discuss applications in various buyer-led SCF techniques and examine a new solution that implements blockchain-based reverse-securitisation. Specifically, they propose the issuing and post-trade clearing and settlement processing of the asset-backed securities that require various intermediaries, data reconciliations and manual intervention to be issued directly into the blockchain as digital assets, thereby switching the ultimate record of ownership from central depositories and custodians onto a blockchain. By doing so they expound an effective and instantaneous clearing and settlement mechanism leading to lower financing costs [69]. Moreover, Li et al. [94] introduce a blockchain use-case in logistics finance to tackle financing shortages for SME retailers. They propose a blockchain-enabled logistics finance execution platform, whereby retailers, suppliers, commercial institution financers and third-party logistics providers can arrange inventory financing by leveraging dynamic pledge of warehouse operations [94]. Du et al. [45] integrate the characteristics of blockchain to solve the problem of non-trust and information asymmetry among the participants in the supply chain and present a solution for warehouse receipts financing through a service platform, which has already been active for a year and has served more than 500 companies in China with an accumulated transaction value of USD 1.2 billion.

**Table 2:** List of articles identified through the research protocol

Theme	Paper	Method	Focus	Areas	Key findings
SCF	Yu et al. [162]	Simulation analysis	Blockchain SCF	Logistics	A blockchain-based financing strategy called Customer Undertakes Guarantee is proposed for logistic-based multi-sided platforms
	Hofmann et al. [69]	Literature review, Conceptual design	Blockchain SCF	Reverse securitisation	Blockchain uses cases are explored specifically in the context of buyer-led payables SCF models to deal with high financing costs and to expedite the existing processes
	Choi [36]	Game theory	Blockchain transactions vs Bank-mediated trade finance	Fashionable products	Comparing the blockchain-based transaction costs with fixed bank-mediated trade credit costs, the blockchain achieves a higher profit and lower operational risk if transaction costs are sufficiently low
	Li et al. [93]	Conceptual design, Case study	SCF solution for SMEs	Advance payment financing, Account receivables, Inventory financing	A frame of blockchain-driven SCF platform is designed and it is theoretically tested against existing SCF techniques with a view to facilitate the implementation of SCF to support capital constrained SMEs
	Du et al. [45]	Case study, Conceptual Design	SCF innovation	Factoring, Inventory financing	A blockchain-based SCF platform was developed and applied to warehouse receipt pledge financing and accounts receivable factoring
	Chen et al. [30]	Case study	Smart contracting in SCF	Auto retail industry	An efficient SCF platform for SMEs in the auto retail industry, which currently serves over 600 enterprises, was documented
	Han et al. [62]	Literature review, Analysis of court decisions	Anti-fraud in SCF and trade finance	International Trade, Export Finance	Discusses the main types of international trade fraud, investigates bank’s due diligence obligations, and suggests practical blockchain-based solutions to curtail trade fraud
	Li et al. [92]	Literature review	SCF risk management with blockchain	Financial Supply Chain	A new SCF risk management method is examined through the establishment of an information sharing blockchain-based platform, which can mitigate credit, legal, market and operational risks
	Chod et al. [35]	Game theory, Blockchain protocol design	Supply Chain Visibility, Inventory signalling vs cash signalling	Loan against receivables, Agriculture	Signalling inventory, which requires supply chain visibility that is provided by blockchain at sufficiently low cost, is more efficient than cash signalling in reducing operational disruptions through less distortion despite higher signalling cost
	Lee et al. [91]	Game theory	Supply Chain Visibility (Information flow inefficiency and Information Asymmetry)	Dynamic trade financing (DTF)	The choice between two trade financing arrangements with uniform interest rates vs dynamic interest rates (DTF) is analysed. Blockchain complements DTF if there are delays in information gathering, while it partially substitutes DTF if there is information asymmetry
Trade Finance	Li et al. [94]	Object oriented methodology (OOM)	Logistics finance	SMEs	A blockchain-enabled logistics finance execution platform has been proposed as an integrated solution to facilitate logistics finance for SMEs in e-commerce retail
	Chang et al. [26]	Multi-case study	Trade finance innovation	Letter of Credit	The integration of blockchain may enhance collaboration among trade parties leading to a paradigm shift towards multiple participant networks
	Chang et al. [25]	Process re-engineering, Object-oriented analysis	International trade transactions	Letter of credit	A blockchain-based L/C process is proposed via the utilisation of smart contract capabilities

The benefits of blockchain in eliminating or reducing information asymmetry have recently been analysed using game theory in Chod et al. [35] and Lee et al. [91]. Based on a signalling game between a buyer and a bank, Chod et al. [35] show that signalling operational quality through larger purchase order quantities leads to less disruptions than cash signalling in the form of inflated loan requests. Inventory signalling requires the bank to verify supply chain transactions, which calls for the use of blockchain. Accordingly, Chod et al. [35] introduce a Bitcoin-based low-cost transaction verification protocol that maintains privacy. The study postulates that a high-type buyer is more likely to adopt blockchain if its reliability increases, if the product has no salvage value, e.g. highly customised or perishable, if its market size increases, and if the verification costs are lower. Focusing on transaction costs, Choi [36] shows that blockchain-based transactions in a newsvendor setting lead to higher profit than a bank-mediated trade, if the blockchain transaction costs are sufficiently lower than the bank charges. Lee et al. [91] compare dynamic interest rates with uniform interest rates in an abstract multi-stage trade finance setting where the bank may benefit from blockchain by reducing the information asymmetry or improving the efficiency of information flows. When there are long delays in collecting reliable information, the blockchain is required for the dynamic interest rates to be rewarding [91]. The academic studies on blockchain SCF are summarised in Table 2.

### Industrial projects and initiatives

The use of blockchain for SCF is being explored by incumbent market leaders as well as start-up companies. Many proof-of-concepts, piloting, or entering production schemes have been developed in the last five years. The purpose of this section is to analyse these newly emerging blockchain projects in trade and supply chain finance and to identify how they enhance existing processes. Table 3 presents a list of popular blockchain-enabled SCF initiatives identified through a practical case-based research on the grey literature.

**Table 3:** List of selected popular blockchain-based projects in supply chain finance

Name	Led by	Objective	Description	Blockchain	Source
We.Trade	IBM and 12 EU-based banks	To facilitate the financial settlement between supply chain partners	We-trade is a ‘bank-centric platform’ that utilises blockchain-enabled smart contracts to guarantee payments and to provide several SCF products, such as invoice financing, and Bank Payment Undertakings	Hyperledger Fabric	[52, 62]
Skuchain	Skuchain	To provide financing to suppliers at the buyer’s cost of capital	Skuchain is a B2B platform which utilises blockchain technology to enhance buyers’ visibility into their inventory and provide SCF solutions at the lowest cost of capital in the chain	Hyperledger	[16, 100]
Chained Finance	Fnconn and Dianrong	To provide suppliers with easier access to SCF solutions	The Chinese P2P lender Dianrong partnered with FnConn, the financing limb of the world’s largest contract manufacturer of electronics (Foxconn), to provide SMEs-suppliers of large enterprises in China with SCF solutions	Corda	[30, 104, 130]
Tradelens	Maersk and IBM	To provide end-to-end visibility to the global supply chain	Tradelens is a blockchain-enabled ecosystem supported by IBM, Maersk, and other major industry players as well as ports and customs authorities aimed at digitalising global trade	Hyperledger Fabric	[77, 78]
Komgo	A consortium of 15 banks and corporates	To streamline trade finance processes	Komgo is a fully decentralised commodity trade finance network which does not only offer digital SCF solutions (including receivables discounting, inventory financing and Standby L/Cs) but also a KYC solution and a certification feature	Quorum	[39]
BAFT DLPC	The Bankers Association for Finance and Trade (BAFT)	To design a legally binding and enforceable payment commitment	DLPC working group released two best practices documents regarding business and technical perspectives. The payment commitments recorded on a distributed ledger can be used in the context of trade finance or SCF as a discounting or advance payment method	Corda and Hyperledger Fabric	[43, 44]
Clipeum	A consortium of 12 EU financial institutions	To build an EU Know your Customer (KYC) and digital identification network	In Clipeum every participant has a vault in which they store their KYC information and they retain full control over data sharing and access permissions by providing access to their counterparts upon requests	Corda	[129, 145]
Contour	R3 along with major banks	To digitalise letter of credit processes	Contour network is the legal entity which commercialises the blockchain trade finance project Voltron. In this project, pilots involving 14 countries have been carried out which reduced the processing time for L/Cs from 10 days to under 24 hours	Corda	[110, 156]
Finacle Trade Connect	Infosys Finacle	To facilitate inter-organisation SCF and trade finance processes in India	Finacle Trade-Connect is a blockchain-based solution available for a range of processes, such as L/Cs, Open Accounts, Bill of Exchanges, PO Financing, Invoice Financing, Bank Guarantee, Factoring and Reverse Factoring	Corda	[49]
Marco Polo	TradeIX	To support working capital finance solutions via a blockchain platform	Since March 2019, SCF transactions pertinent to receivables finance, payable finance and payment commitments have been successfully piloted on Marcopolo	Corda	[132]
Halotrade	Halotrade	To facilitate sustainable SCF solutions	Halotrade provides supply chain visibility that enables buyers and financers to automatically incentivise sustainable production practices	Ethereum	[151]
Hyperchain	A consortium of public/ private institutions	To realise SCF visualisation and reduce the financing cost of SMEs	Based on the secure sharing of information Hyperchain is a Chinese enterprise-level blockchain platform which reduced the financing cost of SMEs relying the credit transmission of the core enterprises	Proprietary	[109]
eTradeConnect	Hong Kong Trade Finance Platform	To provide corporates with easier access to working capital from banks	eTradeConnect has digitised PO and invoice creation, pre- and post-shipment trade finance and payment status updates. Participants benefit from the potential access to multiple banks for supply chain financing	Hyperledger fabric	[152]
TradeFinex	TradeFinex	To be ‘a network of networks’ by providing several trade finance projects	TradeFinex is commercially active with receivables discounting, letter of credit, securitisation, bank guarantees and digital bills of lading applications	XinFin Network	[109, 123]
Ant Blockchain Open Alliance	Ant Financial and Alibaba Group	To provide SMEs with easier access to finance at competitive rates	Ant Blockchain Open Alliance was launched by Ant Financial, the financial limb of Chinese E-commerce giant Alibaba to leverage transparent electronic reporting so that the account payables can be used as credit certificates for the suppliers	Ant Blockchain	[70, 88]
Digital Trade Standards Initiative	ICC	To foster standardisation and inter-operability	DSI was established to develop standard interfaces that will connect existing ‘digital islands’ and enable inter-operability between blockchain-based digital trade projects	N/A	[109]

The findings indicate that reviewed projects can be compiled into categories according to the problems they are trying to solve. For example, We.Trade, Skuchain, and eTradeConnect utilise various business models to enhance existing processes and provide better SCF products through sharing of information and digitisation of the relevant paper-based documentation. Blockchain is also being used under Letters of Credit (L/C) by the Contour network, Financle Trade Connect, and TradeFinex, which are among the most popular trade finance projects in the industry. Similarly, the Marco Polo Network, which consists of 30 banks, aims to facilitate SCF solutions via a DLT-based platform inter alia by providing distributed data storage and bookkeeping, identity management, and asset verification [109]. In this context, the Digital Ledger Payment Commitment (DLPC) provides a payment undertaking in digital form on a blockchain for use in any trade finance transaction, which is legally binding, enforceable, negotiable and independent in a sense that it is not contingent on the underlying trade transaction [43]. Komgo and Clipeum do not only offer digital trade finance-related products, but also Know-Your-Customer (KYC) compliance services which enable the transmission of data stored in a blockchain-based platform among the participating entities on a need-to-know basis [39, 129]. Some projects, such as Chained Finance, Halotrade, Skuchain, Hyperchain and Ant Blockchain Open Alliance leverage DLT to enhance financial transparency of micro, small and medium-sized enterprises (MSME) [151]. Skuchain, specifically, utilises a blockchain system to enhance buyer’s visibility into their inventory and provide better financing to MSMEs by allowing them to get financing at the buyer’s cost of capital, whereas Hyperchain can digitise the accounts receivable, store them in the blockchain, and based on secure information sharing allows MSMEs to benefit from the credit status of the core enterprises, such as large manufacturers. To solve the issue of inter-operability among the various blockchain-based networks and other technology platforms, organisations, such as TradeFinex and the International Chamber of Commerce’s (ICC) Digital Trade Standards Initiative (DSI), are focusing on technical standardisation [109]. An extensive analysis of each identified project is beyond the scope of this study withal. In the following section the review combines information extracted from these projects and the literature to underline how specific features of blockchain technology can address existing inefficiencies in SCF.

## Findings and discussion

This section analyses both the academic literature and blockchain-based SCF projects from the perspectives of (i) pain points and barriers in existing SCF processes, (ii) the promise of blockchain-driven SCF solutions, and (iii) implementation challenges.

### Pain points and barriers in supply chain finance

Considering that blockchain solutions apply to different existing problems, understanding the pain points and barriers in SCF processes is necessary to perceive how blockchain can revolutionise SCF. The analysis of the selected literature suggests that lack of visibility in physical supply chain processes, time consuming and inefficient manual paperwork, regulatory and compliance related costs, the risk of fraud, and high transaction costs are essential barriers in SCF in general.

#### Lack of supply chain visibility

The visibility across the supply chain has been shown to be a crucial requirement for trust, collaboration, and coordination in supply chains, resulting in the stabilisation of material flows, reduction in demand distortion and increased efficiency and agility [12, 29, 51, 133, 161]. For supply chain finance, the end-to-end visibility of financers into the material flows as well as the financial flows from invoice to cash is essential [35, 88]. However, even the biggest corporations lack the capacity to access reliable and up-to-date information throughout their extended supply networks [103, 153]. The principal cause of high financing rates and transaction costs in the incumbent trade and supply chain finance processes is the risk premium due the lack of transparency in credit evaluation processes [65, 93]. Moreover, the limited visibility does not only ignite more than 25,000 disputes in SCF every year with USD 100 million tied up at any given time [15], but also hampers the collection of receivables for the core firm [47, 92]. The lack of visibility impedes trust and commitment among supply chain partners [46, 119] and foments moral hazard problems [34] as well as more general adverse effects of information asymmetry [35, 91], which result in sub-optimal operational decisions that expose stakeholders in supply chains to financial risks [10, 13, 127]. As a result, many actors in the chain operate in opacity and a large group of MSMEs are precluded from SCF [45], especially if they do not transact directly with the core enterprises [93].

#### Laborious and inefficient processing of manual paperwork

The ingrained dependence of SCF on paper-based documentation has driven up costs and caused inefficiencies in SCF [2, 36, 117, 144]. Sequential input and manual checking of the paper documentation is costly and error prone [25, 30], and results in delays in invoice reconciliation as well as in the receipts of payments [103]. Costs occur from the complexity of inter-organisational supply chain collaboration and intra-firm cross-functional coordination [124, 165]. Tedious, time-consuming and opaque document flows that use a computer-paper-computer manual operation model [85] introduce errors and risks [155], resulting in high administrative costs [25] and expensive billing operations [15]. The cost of processing this paperwork is estimated to be between 5 and 10 percent of the transaction value [148].

#### Regulatory and compliance-related barriers

One of the biggest hurdles of the existing SCF processes is the regulatory requirements that have been imposed on financial institutions [74, 103, 117]. According to a survey conducted by the Asian Development Bank (ADB), which investigated the reasons behind the rejection of financing applications by banks, $76\%$ of the surveyed banks highlighted the cost and complexity of conducting Anti-Money-Laundering (AML) and KYC checks as the principal barriers in expanding their trade and supply chain finance operations [1]. Considering that the approval of SCF applications is manual and complex, usually only the most well-known applicants are currently being approved, while MSMEs applications remain under-served [2, 74]. Therefore, AML/KYC compliance procedures increase transaction costs and lower the profit margin, thereby reducing the chances of SCF applications being accepted and causing a shortage of SCF around the globe [62].

#### Risk of fraud

The massive amount of money and documents changing hands in trade and supply chain finance transactions render them susceptible to attack from fraudsters [15, 30, 74]. The risk of fraud can be defined as the possibility that the receivable does not exist or varies from how it is represented [62]. L/Cs, purchase orders, invoices, warehouse receipts, and bills of lading (B/Ls) are all subject to tampering and alteration [14, 98]. Some common types of trade finance fraud are multiple invoicing, over-invoicing, duplicate B/Ls that are financed multiple times, forged B/Ls and L/Cs, and backdating of transport documents [28, 62] or even repeated pledges and empty pledges caused by asymmetric information and adverse selection [25, 93]. Fraudulent trade and supply chain financing deals plague SCF as evidenced by the USD 10 billion uncovered fraudulent deals only in China during the year of 2014 [62].

### Corresponding benefits of blockchain-driven supply chain finance

The barriers and challenges highlighted above have created a need for digitalisation in the SCF sphere. As discussed in previous parts, blockchain integration emerges as the most promising drive towards digitalisation of the SCF processes. Blockchains pledge to streamline the flow of information in supply chains and achieve the synchronisation of material, information, and financial flows [10, 95]. In the following, we analyse the ways the blockchain-driven SCF has been proposed or shown to address the challenges above based on the review of the academic studies and the industry applications summarised in Tables 2 and 3, respectively.

#### End-to-end supply chain visibility

The increased supply chain visibility has been presented as a pillar of blockchain technology [9, 113]. Due to the integrity and immutability of records, blockchain enables real-time trade and cargo information from a single source of truth [92, 150]. For example, Tradelens provides real-time visibility of the progress of goods and documents in the container transportation industry through its blockchain ecosystem [78]. Visibility provides transparency, which is crucial for orchestrating SCF programs [92] as it solves issues of information asymmetry within the supply chain that drive financing costs higher [45, 93]. Since the SCF decisions and premiums are driven by the fluctuation of credit risk [55], information transparency provided by blockchain enables financers not only to view the credit history of the applicant [47], but also to monitor other related operational and financial data, such as order quantities, latest warehouse, shipping, and payment statuses [69], thereby gauging their risk estimations dynamically [91]. The traceability of collaterals in providing SCF solutions is a key benefit distinguishing blockchain ecosystems from other existing platforms [9, 30]. It could also provide an unacknowledged applicant, such as an SME, with the opportunity to evidence its creditworthiness to a financer, thereby securing favourable financing terms with improved operational performance [35, 36].

#### Increased speed and operational efficiencies enabled by digitalisation, smart-contracts, and the Internet of Things (IoT)

The promises to expedite transactional processes and to lower the overall costs of financing bring substantial benefits to all stakeholders involved in an SCF transaction [25]. Hofmann et al. [69] argue that the combination of blockchain with IoT can maintain device connectivity and deliver material flow tracing across the supply network so to adjust the risk premium throughout the shipping process. IoT enables feeding the blockchain with instant information via sensors, rather than having to rely on human ‘oracles’ to transmit data about the physical movement of goods [26]. This application involves using Radio Frequency Identification (RFID) tags, GPS tags, and other chips in the form of installed detectors throughout the physical chain [147, 159] to achieve real-time monitoring and tracking of data [120], which can be leveraged by smart contracts to automate the execution of transactions [93, 149]. The latter constitute automatable and enforceable agreements that can run on blockchains by coding various contractual terms into computer code [24, 134]. Undoubtedly, there is a resemblance between the programmable nature of smart contracts and the state-contingent character of traditional trade finance procedures, such as documentary collections and L/Cs [17]. For example, trade finance techniques, are usually designed to release a tranche by detecting that some pre-determined conditions have been met, such as that a B/L has been sent or that a shipment has been made [25, 159]. The flexibility of smart contracts renders them suitable to automate further SCF solutions, such as receivables or payables finance. Automation is achieved through implementing staged trigger points for key events for a range of SCF solutions [69, 93, 112], resulting thus in efficient, transparent and cost-effective flow of information and value [150].

In practice, numerous initiatives have been vigorously researching blockchain-supported proposals that tackle the inefficiencies occurring from manual processing of information in trade finance (see cases from Komgo to Marco Polo in Table 3). For instance, by utilising a blockchain-based network that links all the entities involved in a L/C transaction, platforms like Finacle Trade Connect and Contour have achieved to reduce the end-to-end processing time by 90 per cent. Similarly, Komgo promotes structured data fields instead of documents in its platform, so that it can streamline seamlessly the entire document workflow in trade finance transactions in its platform. More ambitiously, TradeFinex provides a marketplace for peer-to-peer trade and SCF transactions utilising cryptocurrencies. The BAFT DLPC provides a legally binding digital payment commitment in fiat currency, which can inter-operate with Skuchain to digitalise L/Cs and other trade and SCF transactions and automate execution of these instruments through smart-contracting [156].

#### Reduced regulatory costs

Blockchains constitute distributed trustworthy databases, shared by a community, which can be used for KYC, Customer Due Diligence (CDD), and AML purposes [25, 117]. The key functionality for financers of an immutable ledger, in which near real-time data are recorded, is the provision of reliable evidence about new clients, such as IDs and any relevant background documentation [69, 159]. Process integrity, disintermediation and decentralisation can enable secure information sharing amongst various parties [120], thereby rendering it possible to eliminate duplication of regulatory compliance processes, such as KYC checks, by sharing the existing information on a blockchain so that other financers would no longer need to execute the same controls manually [52, 107]. Blockchain can, thus, enable a system where all financers simultaneously hold KYC data and benefit from economies of scale resulting from checks needing to be undertaken only once [11, 164]. As evidenced in Table 3, some blockchain projects, such as Clipeum or Komgo, are building platforms where the members can upload KYC documents and authorise other participants to consult these documents upon request on a need-to-know basis [39, 66]. Therefore, blockchain could assist in credit checks, diminish compliance costs, and, thus, simplify the establishment of SCF programs.

#### Mitigated fraud risk

As explained in Han et al. [62] and Lawlor [90], the primary aim of tokenising trade documents on a blockchain is to avoid fraud and double-financing issues. As an immutable and shared registry [150], blockchain can preserve the integrity and authenticity of the trading background, including shipping and warehouse status and purchase order data, which are vital for SCF techniques [93]. Each document is hashed and time-stamped to create an original identifier, and, if a malicious actor attempts to use the same document for financing purposes through the platform, that identifier signals the previous case of financing to all parties [69]. Thus, blockchains limit forgery and multi-financing issues in, for example, inventory financing, pre-shipment financing, advance against receivables and distributor finance techniques [69], thereby enhancing SMEs credibility to obtain financing from previously hesitant financers [94].

### Implementation challenges to further adoption of blockchain technology in the SCF sphere: toward a more collaborative business model?

Thus far, this paper discusses how blockchain technology can transform trade and supply chain finance processes. This section reveals the challenges associated with blockchain implementation in this environment, which are summarised in Table 4.

**Table 4:** Implementation challenges for blockchain adoption in supply chain finance

Category	Key elements	References
Inter-Organisational	Opposition from incumbents	Du et al. [45]
	Reluctance to share private information	Korpela et al. [85]
	Standardisation and inter-operability of business processes	Kouhizadeh et al. [86]
	Supply chain coordination through a business model that leverages blockchain along the supply chain	Michelman [101]
		Saberi et al. [120]
		Van Hoek [147]
		Wang et al. [149]
Intra-Organisational	Factors that affect managerial decisions on IT adoption	Bogucharskov et al. [17]
	Uncertainty about the value and use of the technology	Iansiti and Lakhani [71]
	Cost of transforming and managing legacy systems	Kamble et al. [80]
	Cultural hurdles against innovations	Kouhizadeh et al. [86]
		Queiroz and Fosso Wamba [115]
		Saberi et al. [120]
		Van Hoek [147]
		Wang et al. [150], Yang [159]
Technical	User-friendliness	Babich and Hilary [9]
	Energy consumption	Chang et al. [27]
	Scalability	Kouhizadeh et al. [86]
	IT security	Kshetri [87]
	Immaturity of sensor devices	Lu and Xu [97]
	Data quality	Wang et al. [149]
		Zamani et al. [163]
Legal	Legislative requirements of paper-based bills of exchange and promissory notes	Batwa and Norrman [15]
	Uncertain legal status of digitalised documents of title	De Filippi and Wright [41]
	Legal enforceability of smart contracts	Goldby [57]
	Allocation of risks in decentralised platform	Kouhizadeh et al. [86]
	Synchronicity between the state of the blockchain and the legal status	R3, Shearman & Sterling LLP, BAFT [116]
	Jurisdictional issues	Schuster [125]
		The Economist [138]
		Wang et al. [150]

#### Business implementation challenges

A decentralised and immutable database which enables SCF stakeholders to securely share peer-to-peer digital trade documentation and tokenised assets entails a paradigm shift toward automation, real-time risk management, and cheap, efficient, and inclusive financing at reduced administrative cost [9, 26]. However, there is evidence of opposition from incumbent economic leaders within the banking system to the blockchain transformation in SCF out of fear of being cut-off [149] or of missing revenue streams [101]. Other actors are unwilling to share valued information and reluctant to the total transparency provided by blockchain [82, 149]. Given that production costs, order quantities and transaction prices are usually perceived as trade secrets, privacy concerns will be a major problem in SCF should visibility be achieved [45]. Hence, parties that extract information rent are expected to be reluctant to take part in blockchain platforms that decrease information asymmetry.

Saberi et al. [120] analysed inter-organisational blockchain implementation challenges, alongside intra-organisational, system related, and external to the supply chain challenges. They identified information sharing issues, cultural differences, and challenges in coordination and communication that impede collaboration in supply chains [120]. Kouhizadeh et al. [86] detect the complexity of blockchain technology and the need for re-engineering of business processes across the supply chain in an orchestrated manner as the inter-organisational barriers, in addition to the aforementioned confidentiality and security concerns. Korpela et al. [85] focus on the requirements for the digital supply chain transformation to succeed. Companies must develop their business model to maximise effectiveness in leveraging blockchain in their business offerings and should establish information model platforms to achieve inter-operability and integration among multiple internal platforms of various organisations [85]. As discussed in supply chain collaboration literature based on EDI, CPFR, and RFID technologies [50, 114, 122], the industry must develop standards which would enable business-to-business (B2B) process connectivity so that members in SCF transactions can exchange original documents and conduct transactions online [147]. Lastly, integration channel intermediaries, similar to EDI or SWIFT operators, are needed to reconcile data formats and distribute information across the various blockchain systems of independent organisations [85]. In this regard, several industrial projects (e.g. TradeFinex and Digital Trade Standards Initiative in Table 3) explicitly refer to the need for standardisation as a prerequisite to utilise blockchain in SCF.

#### Managerial implementation challenges

Despite that blockchain provides for networked applications across an ecosystem of companies, with no single party controlling the application [142], to ensure that a company’s systems are compatible with blockchain SCF platforms requires surmounting some managerial challenges. Batwa and Norrman [15] discovered that the lack of acceptance in the industry, lack of technological maturity, and the need for collaboration and coordination among competing parties are the main obstacles for blockchain integration in SCF processes. Likewise, Queiroz and Fosso Wamba [115] discuss implementation challenges through the prism of technology acceptance models in order to understand the individual behaviours in IT adoption based on performance expectancy, effort expectancy, facilitating conditions, perceived usefulness, and trust among supply chain actors. Other scholars suggest institutional theory, diffusion of innovations theory [118], theory of planned behaviour, technology readiness and the classical technology acceptance model [80] to explain the reasons why a particular organisation adopts a new and disruptive technology [86, 150, 159].

In this context, Iansiti and Lakhani [71] developed a blockchain applicability model based on how innovative technologies are naturally being adopted. To this end, Wang et al. [150] propose using sense-making in assisting managerial decision-making, which refers to the process of developing specific assumptions, expectations, and an awareness of the said technology [147], which then frame the actions of the decision makers towards it [99]. Wang et al. [150], thus, focus on managers’ prospective sense-making perspectives and extricate their views on the issues that may negatively influence blockchain diffusion through interviews with 14 supply chain experts. Numerous stakeholders who may develop conflicting objectives would be involved in a blockchain platform. Therefore, cultural hurdles against new innovations, data ownership and intellectual property issues, the lack of standards, costly implementation, security issues and regulatory uncertainties present barriers to blockchain deployment in SCF [120, 159]. In this regard, a solution to overcome these challenges has been suggested arguing that government-led initiatives and a paradigm shift toward a more collaborative business model in the industry could convince top management in organisations aboard blockchain SCF platforms [150].

#### Technical implementation challenges

Lu and Xu [97] and Kouhizadeh et al. [86] discuss technical issues, such as usability, energy consumption, size and bandwidth and throughput latency, while Wang et al. [149] point out that despite the immutable character of blockchain, hacking is still possible [163]. In a similar fashion, Kshetri [87] highlights the technological immaturity of sensor devices, the borderline between the physical and virtual worlds, and the high degree of computerisation that might not be accessible in some parts of the world. Moreover, Babich and Hilary [9] underline the ‘garbage in, garbage out’ weakness, namely the issue that there might be discrepancies between the information recorded in the blockchain and the physical state due to mistakes or intent. Although IoT is often presented as the solution to the flaw of introducing erroneous data into the blockchain [27], the technological risks of the system are not sufficiently discussed in the extant literature. For instance, the system is vulnerable to fraudulent activities by malignant actors, who may separate the sensor from the rest of the cargo to automatically trigger the release of an unlawful payment.

#### Legal implementation challenges

Despite the continuous development and improvement of the technology to achieve digitalisation in SCF, the absence of enabling regulatory and legal frameworks and broadly accepted standards may impede blockchain diffusion in SCF. For example, both Article 3 of the Uniform Commercial Code (UCC) in the US and its ancestor, Article 3 of the English Bills of Exchange Act 1882, apply only to ‘written’ bills of exchange and promissory notes, thereby not covering bills of exchange, promissory notes, and other negotiable instruments or payment commitments that are in digital form and registered via blockchain in SCF [116]. Similarly, the market practice in international trade is currently dependent on paper negotiable bills of lading and other paper documents of title [138] as there is uncertainty regarding the legal value of digitally issued documents of title [57].

Other legal issues relate to the legal enforceability of smart contracts and to the legal liabilities of decentralised blockchain platforms, with respect to whom is responsibility attributable for platform-related risks, such as system malfunctions, leakage of sensitive information, insuring against risks and non-compliance with regulations, including data protection regulations [41]. Further legal issues that need to be addressed include the legal status of blockchain records and the issue of synchronicity between the state of the blockchain and the legal status, which might be different due to the occurrence of fraud or incapacitation [125]. The situation is further complicated as blockchain-driven SCF operates worldwide, which requires numerous parties to comply with different national laws, regulations, and institutions [61].

Current solutions rely on private legal frameworks established through multipartite agreements-contracts to establish rights and liabilities [57]. However, without coherency and unification, the market is vulnerable to fragmentation. Hence, the adoption of a stable legal environment is imperative for blockchain-based trade and supply chain finance to succeed [15, 150]. Even though the SCM literature does take into account legal considerations in abstract, as a general factor that impedes blockchain adoption in SCF [86, 150], there is limited in-depth consideration of the specific legal issues that arise and affect the feasibility of each theoretical proposition.

### Critique and future avenues of research

Building on the proposition of Saberi et al. [120] that supply chain governance mechanisms must be further evaluated for effectiveness in understanding blockchain-based supply chains, it is argued herein that future research should integrate some overlooked analytical frameworks and employ empirical methods as well as mathematical modelling in order to investigate blockchain implementation challenges further and propose solutions.

#### Global supply chain management

According to the idealised view of supply chain management, supply chains are perceived as networks of organisations that collaborate together to produce competitive advantage [37]. However, firms might get stuck in long-term adversarial relationships with their suppliers, making them susceptible to opportunistic behaviour due to information asymmetry [83]. As discussed above, blockchain promises to address this issue by ensuring trust through immutability of records and transparency. However, this necessitates the participation of the stakeholders in the first place. Mechanisms for incentivising blockchain participation remains a major strategic challenge and an open research question [124]. Therefore, further theoretical development is needed to understand the conditions for the establishment of blockchain-based SCF networks.

#### Platform theory and strategic management

We suggest that blockchain SCF networks may be conceptualised as ecosystem platforms [76], which consist of members that are themselves other organisations and operate as evolving organisations or meta-organisations [3] that shift along a continuum of different innovation configurations [53]. This means that potential innovators of complementary products can utilise Application Programming Interfaces (APIs), to build compatible complements [40]. As Google or Facebook have developed and shared APIs to encourage independent software developers to build applications [53], blockchain platforms can provide the necessary open APIs in the form of flexible script code system to encourage participants to code smart-contracts and offer innovative payment and financing solutions [93]. For instance, companies may promote Tradelens and create complementary services, such as smart contracts and other decentralised SCF applications, on top of its platform for their clients. This may enable new SCF channels, such as an open market for financing of invoices [47]. To this end, Choi [36] reported new blockchain-enabled SCF solutions in which participants conduct transactions peer-to-peer using cryptocurrency and concluded that these solutions can yield higher expected profits and lower level of operational risk compared to existing SCF techniques.

#### Co-opetition strategy

The current debate regarding the appropriate strategies and operational practices for the use of blockchain in SCF can be improved by drawing on the co-opetition strategy that combines competition and cooperation to leverage on the shared resources [18, 154]. As we have seen in Table 3, most of the projects trying to leverage blockchain technology within the financial supply chain sphere are essentially consortia. Being a network-based endeavour, blockchain technology is facilitating cooperation between competitors. In this regard, a V-form organisational structure has been suggested as ‘an outsourced, vertically integrated organisation’ tied together by blockchain [5]. This form of organisation is comprised of an ecosystem of fully independent companies which coordinate and audit their activities through DLT [41, 79]. Future research could focus on the notion of co-opetition with a view of determining the organisational conditions under which a blockchain SCF network is feasible and stable. Game theoretical network formation models [75] provide an analytical framework for such an endeavour, and can help identify SCF methods, market structure, and economic conditions under which blockchain-based SCF can be established.

#### Legal analysis

Another promising direction of research is the articulation of the legal implementation challenges, which is already underway by one of the authors. For instance, the lack of a sufficient legislative and regulatory framework for blockchain alternatives to paper trade documentation begets a risk of a legal void surrounding the use of blockchain SCF platforms. The key legal issues raised by the development and the use of blockchain records operating on global trade platforms need to be explored by legal scholars in order to establish how would the legislative and regulatory environment need to change to ensure legal enforceability of blockchain-based SCF solutions.

#### Information systems and empirical analysis

Further studies could investigate the underlying technology in more depth. For example, a comparative study regarding the appropriateness of different blockchains of the same type (e.g. Hyperledger Fabric and Corda) for SCF would be an important contribution. Currently, most academic studies investigate blockchain and SCF by utilising either conceptual or simulation methods. Future studies should consider more mathematical modelling and empirical studies to develop an analytical understanding of the key factors that drive the relationships between different types of flows and stakeholders with conflicting interests acting on networked systems. For instance, there has been little empirical investigation into the blockchain impact on terms of return on investment and realised customer value [143] or on its impact on critical supply chain properties, such as network risk and resilience.

### Limitations

Finally, a few limitations of this literature review need to be considered. First, this review focuses on the impact of blockchain technology on SCF. The authors acknowledge that the choice of keywords might have excluded some relevant blockchain articles. Here, we aimed to provide a concise discussion of the implications of blockchain in trade and supply chain finance, while a comprehensive discussion on the broader benefits and challenges of blockchain for SCM is beyond the scope of this article and has been provided elsewhere [27, 113, 115, 149]. Second, most industrial projects are at their early stages; hence, there is limited empirical data on the results of these projects. Thus, the conclusions have to be drawn from the analysis of the projects based on restricted information in the public domain as well as theoretical discussions in the literature. Third, the academic literature on blockchain-enabled SCF is in its infancy and the publications are dispersed over journals in various fields and topics. This review provides a starting point for future studies that may quantify the significance of the various implementation challenges, identify causal relationships among them, and suggest possible solutions to effectively manage blockchain adoption in trade and supply chain finance.

## Conclusions

The current pandemic has made clear that digitalisation and platform-enabled change is the only way forward for international commerce [106, 126]. It forced corporations and banks to digitalise their operations, seek digital alternatives to wet-ink documentation and understand the inefficiencies of the existing internet solutions and internal systems [72]. This crisis might evolve into an opportunity for the industry to acknowledge the need for creative technology solutions, and to invest in and embrace blockchain resources toward digitalisation [73].

This review contributes to the SCF literature by articulating the rationale behind blockchain adoption. It has enriched this emerging field by discussing several theoretical studies and industry blockchain applications. This paper is one of the first to consolidate the state-of-the-art of blockchain applications in trade and supply chain financing. By elucidating the current perspectives in academia and practice, the areas where blockchain may bring value to trade and supply chain finance have been identified. This review sets out to explore how blockchain technology may transform SCF by exploring the answers to three research questions.

The first research question (RQ1) concerned the key barriers and pain points that hold back innovation in existing SCF processes and contribute to the growing financing gap of international commerce. Our literature review found that the lack of visibility into supply chain material flows, the inefficient manual processes, the paper-based documentation, the burden of compliance with regulations, and the risk of fraud are the main bottlenecks in existing SCF processes. The second research question (RQ2) probed how blockchain combined with related technologies, such as smart-contracts and IoT, can provide solutions to these inefficiencies. Via an analysis of the academic literature, grey literature, and blockchain use cases, the expected gains from blockchain adoption in trade and supply chain finance were identified to include the provision of end-to-end supply chain visibility, the increased operational efficiencies, the reduced transaction and regulatory costs and the mitigation of fraud-related risks. Our review allowed us to further capture several blockchain implementation challenges in SCF at the frontier of practice, ranging from business and managerial implementation challenges to technical and regulatory issues, which were the focus of RQ3. On this question the research attempted to introduce a novel viewpoint in the discussion, suggesting that future academic literature can examine blockchain adoption challenges in SCF through game theoretical models and using the concept of co-opetition, which is tailored to blockchain platforms wherein many competing companies participate and collaborate.

To our knowledge, this study is one of the very few to have contemplated the implementation challenges for blockchain adoption in SCF. It brings valuable insights about SCF and blockchain, thus placing a foundation to motivate further cross-disciplinary research on this emerging technology and range of financing solutions. It will also help practitioners to further understand where and how blockchain may revolutionise SCF processes and stimulate managers to develop strategies and employ the necessary changes that are required for blockchain-driven SCF to succeed. Considering the nascent nature of the technology, regulators can either instigate and mould the development of blockchain-based SCF solutions through pro-innovation policies and regulations or constrain their impact by strict over-regulation. Therefore, understanding how to regulate blockchain-based projects presupposes an analysis of its novel use-cases [41]. Our review provides such an analysis of blockchain-based solutions in trade and supply chain financing, along with a state-of-the-art examination of the theoretical solutions, thus enhances the ability of the regulators to identify further legal issues that might emerge and design laws and mechanisms that will facilitate innovation.

## Figures and Tables

**Fig. 1:**
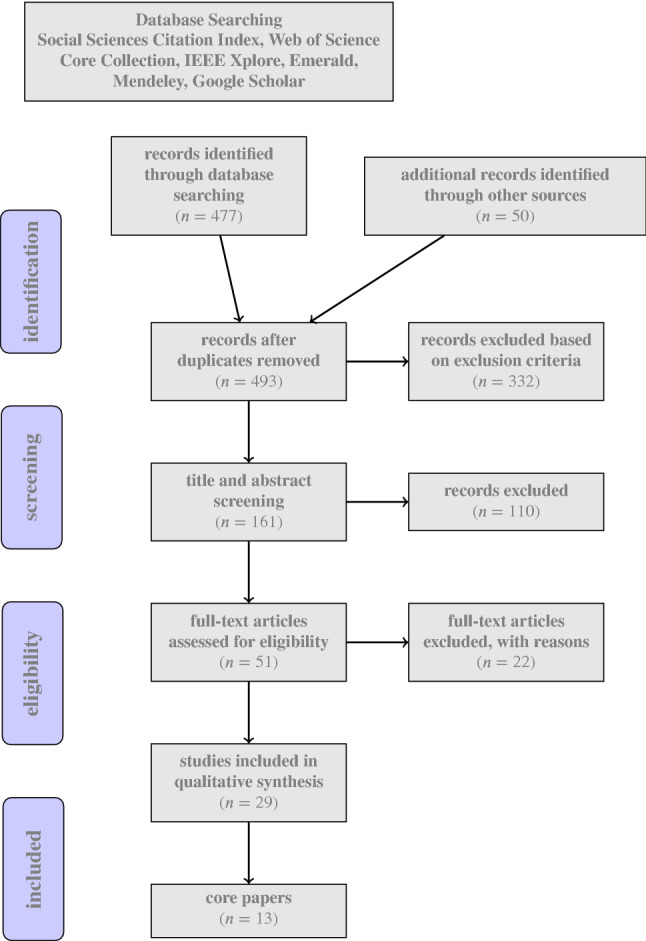
Procedural steps of the search protocol for the academic literature, Source: Moher et al. [102]

## Data Availability

Not applicable.

## APPENDIX: Materials and methods

Considering the rapidly evolving nature of blockchain technology and the paucity of publicly available results on the implementation of blockchain-supported SCF, we have used critical literature review methodology to be able to generate new perspectives [140]. To conduct a transparent and reproducible critical literature review, the process suggested by Torraco [140] and Snyder [128] has been adopted, which was extended by some elements of the PRISMA statement (see Fig. 1).

The review covers the state-of-the-art use of blockchain in SCF in the past five years, 2016 to 2020. Primary data is collected through a systematic search and review of the literature [59], while additional data is collected from grey literature. To avoid biases stemming from omitted literature, the articles were located through keyword search in the core collection of Web of Science of terms related to SCF, trade finance, and more generally, supply chain and international trade. Considering the inter-disciplinary nature of the topic and the diversity of the outlets, no constraints were imposed on specific fields or journals. Additional papers were identified through the bibliography of the relevant articles found by the initial keyword search. Finally, the so-called ‘grey literature’ and reports commissioned by public institutions were also examined to capture the current state of industrial applications, which were located through searches in Google and Google Scholar, supplemented by insights gained by attending several industry events and virtual presentations organised by the International Chamber of Commerce (ICC), the World Trade Organisation (WTO), the International Trade and Forfaiting Association (ITFA), the Bankers Association for Finance and Trade (BAFT) and other organisations and industry associations over the summer 2020, in which market leaders discussed their current efforts.

### A.1 Keyword selection

A research protocol was created to search for all relevant papers on the topic and closely related areas. The terms used in the final selection were determined after some pilot searches, where multiple possible combinations of search strings and keywords were tested. After this iterative trial and error process, the search protocol was formulated as shown in Table 5.

**Table 5:** The search string and keywords selection

Category	Keywords
Blockchain	Blockchain*<OR>“Distributed Ledger”<OR> “Smart Contract*”<OR> “Decentrali?ed Ledger”
	<AND>
Supply chain, trade, and platform	“Supply Chain*”<OR> Trade<OR> Document*<OR> “Bill of Lading*”<OR> Ship*<OR> Invoice<OR> Warehouse<OR> Import<OR> Export<OR> Platform*<OR> Ecosystem*
	<AND>
Finance	Financ*<OR> Receivables<OR> Payables<OR> Discounting<OR> Factoring<OR> Forfait*<OR> “Letter of Credit”<OR> “Bank Payment Obligation”<OR> “Open Account*”

As our topic consists of three elements (i.e. blockchain technology, supply chain, and finance), three groups of search terms were included to ensure that all three aspects are fully captured. We included not only the term blockchain in the first group, but also related concepts, such as Distributed Ledger Technology (DLT) or smart contracts, which are sometimes used interchangeably. To narrow down the scope to supply chain processes and international trade transactions, the second group consisted of supply chain and platform-related terms, including keywords such as ‘supply chain’, ‘trade’ and ‘ecosystem’. As the majority of these keywords can be applied in different themes, they were combined with a third string of keywords consisting of finance-related terms. That way, the second group should always be related with both blockchain technology (first group) and financial perspectives (third group). Specific SCF solutions, ‘factoring’, ‘forfaiting’, ‘discounting’, ‘receivables’ and ‘payables’, as discussed in Sect. 2, were also included among the finance related terms. Finally, main trade finance methods and payment mechanisms used in international trade transactions, such as ‘letter of credit’, ‘open account’ and ‘bank payment obligation’, were added. This literature could not be neglected in the present review, because trade finance is not only highly related [54, 160] but it also partially overlaps with the concept of SCF [21, 84]. Consequently, these keywords were searched for in the scientific article titles, abstracts, author’s keywords, and the keywords-plus field.

### A.2 Article selection criteria and process

After employing the above-mentioned research protocol, 493 studies were returned by the keyword search. Specific exclusion criteria were then applied to identify the directly relevant articles. Articles that were written in any language other than English, editorials, calls for papers, book reviews, articles with missing abstracts, and preliminary studies were excluded to ensure transparency, validity, and academic rigour [128]. Moreover, articles for which the focus fell fully under disciplines other than economics, finance, law, and business and management, e.g. computer science or electrical engineering, were removed. In addition to peer-reviewed academic journals, the search included the proceedings of leading international conferences. Furthermore, certain popular books and book chapters on blockchain, such as Chuen and Deng [38], De Filippi and Wright [69], Hacker et al. [41], Hofmann et al. [61] were included to better understand how blockchain is framed within the popular literature. Consequently, 161 studies were obtained.

Following the guidelines of Snyder [128], the literature review can be conducted in phases by reading abstracts first, making selections, and then reading full-text articles, before making the ultimate selection. Papers that discuss mainly different topics, e.g. cryptocurrency markets and Bitcoin’s price fluctuations, or that focus solely on specific sectors, e.g. use of blockchain in healthcare were discarded. As illustrated in Fig. 1, 51 research papers were retrieved and downloaded.

A full text analysis for finer selection of the candidate papers was employed to align the content of the selected papers with the focus of the review. Twenty-two papers were removed from the poll as they were not directly associated with blockchain implementations in SCF. Publications that discussed features of blockchain that support explicitly SCF received further scrutiny pursuant to their relevance, quality and academic rigour. Ultimately, the selected corpus of core publications consisted of 13 records, which are summarised in Table 2 and discussed in Sects. 3 and 4 of the main text.

As academic studies tend to fall behind the practical implementation of technological innovations, relying merely on academic literature would give a rather constringed view of the topic, especially considering the industry is teeming with blockchain projects. Therefore, the above list is supplemented by a desk-based research on blockchain-supported projects and an analysis of documents beyond academic publishing, such as industry-produced research, to provide a solid overview for understanding how blockchain technology is practically being used in SCF. This led to the identification of the 16 blockchain-based SCF projects discussed in the paper.
